# Beyond one-shot nudges: the effects of repeated interventions for healthier online meal ordering

**DOI:** 10.3389/fnut.2026.1787724

**Published:** 2026-05-04

**Authors:** Ildiko Krisztina Preiner, Eyal Peer, Roni Lotan

**Affiliations:** 1 Department of Psychology, Faculty of Social Sciences, The Hebrew University of Jerusalem, Jerusalem, Israel; 2 School of Public Policy, The Hebrew University of Jerusalem, Jerusalem, Israel; 3 For a Change, Research Lab, Tel Aviv, Israel

**Keywords:** choice architecture, digital nudges, food choices, healthy eating, online ordering

## Abstract

Digital nudges offer a promising approach to promote healthier food choices in online meal ordering, but their effects over repeated exposures and across multiple decisions are undertested. We explored how two nudges, Just-in-time feedback and Assortment, individually and in combination, may affect healthy food choices in a three-day experiment using a mock online meal ordering platform. Participants (*N* = 154) were randomly assigned to Feedback, Assortment, Combined, or Control conditions and completed daily lunch orders hypothetically, each including a main dish, side dish, and drink. Across conditions, healthy ordering increased from Day 1 to Day 2 before declining in the Control and Feedback groups by Day 3. The Combined condition, integrating both structural (Assortment) and moment-of-choice (Feedback) approaches, successfully attenuated this decay, resulting in a 24% higher composite health score compared to Control at Day 3 (*d* = 0.66, *p* = 0.04). Component-level analysis revealed that while main dish choices converged across conditions by Day 3, the sustained effectiveness of the Combined nudge was driven by improvements in side dish and drink selections. These findings highlight the potential of multifaceted digital nudges to sustain healthier choices over time in online food environments.

## 1 Introduction

Online-to-offline (O2O) food delivery platforms have transitioned from a niche service to a major global industry over the past decade (1). In the economic literature, the rise of these platforms is often analysed through the lens of utilitarian value, particularly the monetary value consumers place on the time saved by outsourcing meal preparation (2). While this convenience drives market expansion across diverse demographics — including low-income and food-insecure populations (3) — it also highlights a tension between utilitarian efficiency and hedonic motivations that can favor immediate gratification over long-term health (4). Indeed, the proportion of unhealthy items on O2O platforms was found to range from over 50 to 80 percent of total available items (5–8), and unhealthy foods and beverages are more often promoted by these platforms than healthy ones (9). As unhealthy food cues are more influential on food choices than healthy cues (10), O2O platforms may shift user preferences towards less healthy meals. Frequent platform use is indeed associated with greater fast-food and sugary drink consumption (11), and a greater likelihood of living with obesity (12, 13). These trends highlight the need for scalable interventions that help users make healthier choices in online ordering contexts.

Nudges—subtle, choice-preserving modifications to the decision environment—are well suited to this challenge. Extensive research shows that nudges can improve dietary choices in both offline (14, 15) and digital food environments (16–20). Yet despite growing interest in digital nudging, little is known about how these interventions perform across multiple, repeated ordering occasions as most existing studies examine only a single ordering event; a critical question given that online food purchases are typically made habitually, often daily (21, 22). Understanding whether nudges maintain, strengthen, or diminish their effects over time is essential for evaluating their real-world applicability and long-term public health impact (23).

Among digital nudges, two approaches show particular promise in mitigating the prevalent unhealthy bias embedded within O2O platforms. *Just-in-time feedback* interventions provide immediate information about the healthfulness of foods as users select them (24, 25). By highlighting nutritional healthiness at the moment of choice, such feedback can shift preferences toward healthier options (26, 27). Choice architecture, in contrast, structures the decision environment to favour healthier selections by shifting the balance of available options rather than relying on users’ attention or momentary motivation. A prominent example is *Assortment nudges*, which specifically increase the proportion of healthy items relative to unhealthy ones in the menu — reversing the typical unhealthy-majority composition of O2O platforms — and have been shown to improve the odds of selecting healthier meals (28) and reduce caloric intake (29).

Because of their complementary mechanisms—one providing moment-of-choice information to guide individual decisions, the other restructuring the choice environment—the combined use of both approaches may yield stronger and more consistent improvements in food choices than either nudge alone (1, 22). Yet no study to date has directly compared their individual and combined effects over time in an O2O meal-ordering context. This gap limits our understanding of how digital nudges perform in the real-world, repeated decision settings in which they are intended to operate.

To investigate whether nudges effectively increase the healthiness of online meal orders over time, we conducted a 3-day repeated measures experiment involving a mock online meal delivery platform in Israel. Participants were randomly assigned to one of four conditions: Feedback, Assortment, Combined (both nudges), or Control (without nudge). In all four conditions, a “Good For You” label marked the food items that were previously rated as healthy by a registered dietitian. To ensure that participants perceived the healthiness of items similarly, ratings were verified by a pre-survey involving a participant sample that was excluded from this study. To eliminate price effects, all meal components were priced identically (mains at a fixed price; sides and drinks at no additional cost). The main dependent variable of the study was the overall health score of the ordered lunch, calculated for each day of the study. The mixed model design enabled measuring changes in health scores between conditions and days, and thus comparing the effectiveness of different nudges over time. We expected that all nudge conditions would outperform the Control condition.

## 2 Method

### 2.1 Participants

The final sample consisted of 154 adults (71 female, 83 male). Participants were recruited via a professional Israeli recruitment panel and were compensated in the amount of 10 NIS per day for participating in at least two days of the study. Of the total sample, 125 participants completed all three study days, and 29 completed two consecutive days. Participants who reported food allergies or eating disorders, did not identify as omnivores, and ordered lunch online less than once per month (*n* = 36) were not included in this sample.

The final sample size (*N* = 154) was determined based on recruitment feasibility. A sensitivity analysis conducted using G*Power 3.1 indicated that this sample provides 80% power (*α* = 0.05) to detect a small-to-medium effect size (*f* = 0.12) in our mixed-model design, with a critical F of 2.12. Linear Mixed-Effects Models were utilized specifically to retain data from the 29 participants who completed only two of the three study days, maximizing the use of all available observations.

### 2.2 Design

Participants were invited to complete a lunch order on our mock meal delivery platform once per day across three consecutive days between 12–2 p.m. This duration was chosen to investigate the immediate temporal stability of nudge effects while maintaining high participant engagement and minimizing dropout, which often complicates longer-term digital interventions. Participants were randomly assigned to one of four conditions: Feedback, Assortment, Combined (both nudges), or Control (without nudge). To confirm the effectiveness of randomization, balance checks were performed across the four experimental conditions. There were no significant differences between groups in terms of gender (χ2(3) = 4.98, *p* = 0.173), age (F(3,150) = 0.27, *p* = 0.849), or habitual online ordering frequency (χ2(6) = 6.91, *p* = 0.33). These results indicate that the groups were comparable at baseline regarding key sociodemographic and behavioral characteristics.

The Feedback nudge displayed the proportion of healthy items relative to all items in their basket every time they added or removed an item. For example, selecting a healthy main dish together with an unhealthy side dish and drink generated a display indicating that 33% of the basket was healthy, and 66% unhealthy. The Assortment condition presented a menu in which the proportion of healthy items in the main dish and side dish categories was 2/3, as opposed to 1/3 in the Control condition (22). Following each order placement, participants reported on their current mood and hunger levels.

### 2.3 Measures

Each lunch order consisted of three components: a main dish, a side dish, and a drink. We employed differentiated scoring scales to increase measurement sensitivity to incremental behavioral shifts. Main dishes included options that were either healthy (e.g., salmon fillet) or unhealthy (e.g., sausages). Drinks had options that were either healthy, medium or unhealthy, and side dishes had options that varied from healthy, mid-healthy, mid-unhealthy and unhealthy (Supplementary Table 1). This allowed us to detect movement toward relatively healthier options even when participants were not yet willing to switch to the most healthy category. Health scores were calculated separately for each meal component and summed to yield a daily composite health score. To ensure each component contributed equally to the composite, scores were placed on comparable scales anchored at 1 (unhealthy) and 4 (healthy). Main dish healthiness was coded as binary (1 = unhealthy, 4 = healthy). Side dish healthiness was scored on a four-point scale (1 = unhealthy, 2 = mid-unhealthy, 3 = mid-healthy, 4 = healthy). Drink healthiness was scored on a three-point scale, with values of 1 (unhealthy), 2.5 (medium), and 4 (healthy), preserving the same endpoints while reflecting the intermediate category. The resulting daily composite score ranged from 3 to 12, with higher scores indicating healthier meal choices.

## 3 Results

### 3.1 Health scores across days and conditions

**Figure 1:**
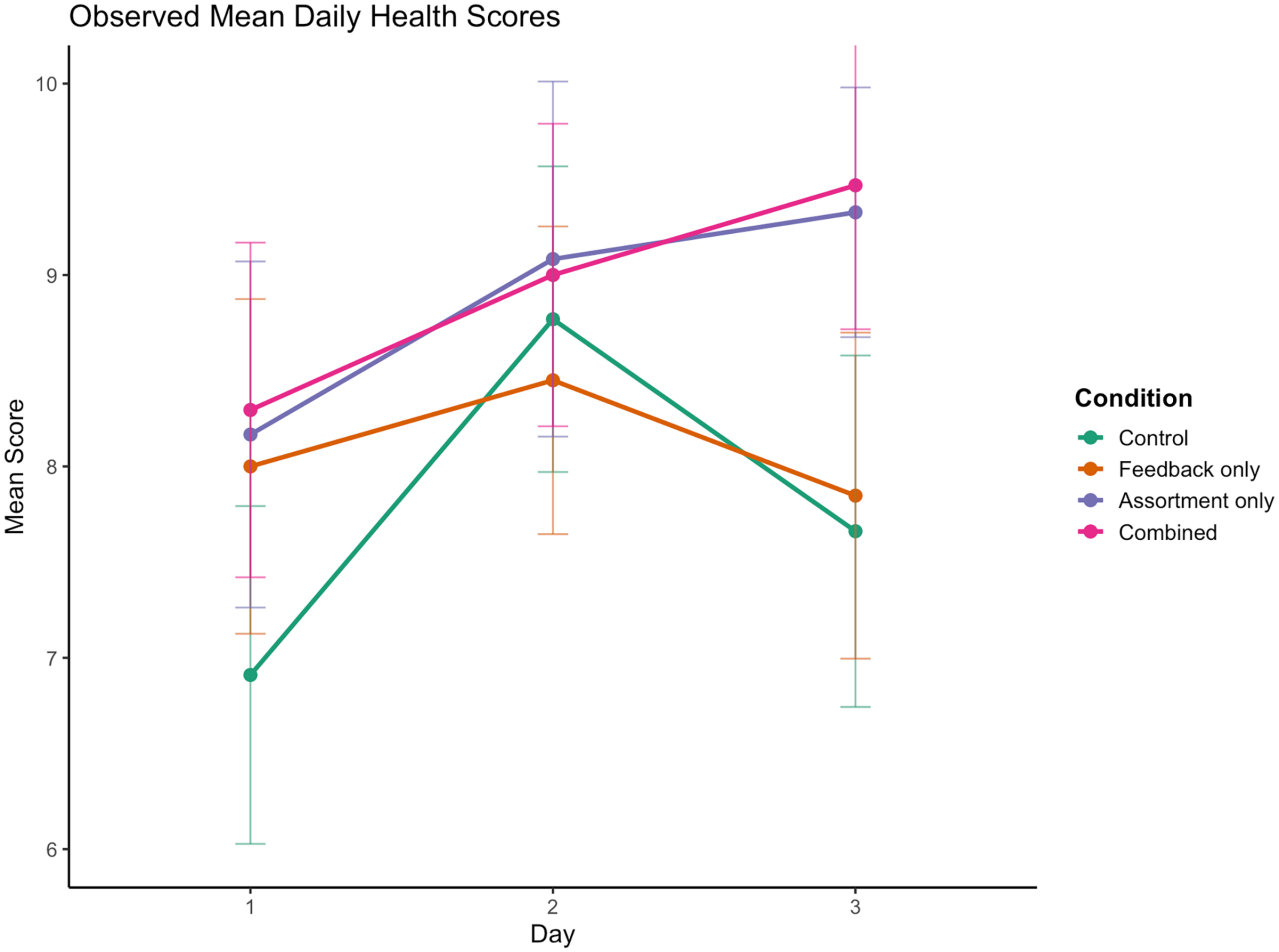
Observed mean daily composite scores per condition. Daily composite health scores were calculated by summing the health ratings of the three meal components selected each day: main dish (binary: 1 or 4), side dish (4 levels: 1–4), and drink (3 levels: 1, 2.5, or 4). This resulted in a theoretical composite range of 3 to 12, where higher scores indicate healthier choices. Error bars represent 95% confidence intervals.

Figure 1 displays the mean daily composite health scores for each condition across the three study days. On Day 1, all nudge conditions scored higher than Control, though only the Combined condition did so significantly (*t* = 2.185, *p* = 0.032, *d* = 0.50). Composite health scores then increased from Day 1 to Day 2 across all conditions, before diverging on Day 3. In the Control condition, scores decreased by 12.7% from Day 2 to Day 3 (*d* = −0.36, *p* = 0.053), while the Feedback condition followed a similar pattern with a 7.1% decline on the final day (*d* = −0.17, *p* = 0.311). Conversely, the Assortment condition showed a non-significant increase of 2.8% from Day 2 to Day 3 (*p* = 0.505), and the Combined condition increased by 5.2% during the same interval (*p* = 0.436). By the end of the study, the Combined condition reached a meal healthiness level 37% higher than the Control condition’s Day 1 starting point (*d* = 0.98, *p* < 0.001) and was 24% higher than the Control condition at Day 3 (*d* = 0.66, *p* = 0.04). To further analyze the composite health scores, we employed a Linear Mixed-Effects Model (LMM) with Participant ID as a random intercept. Condition and Day were entered as fixed effects, with Day treated as a continuous variable to allow for the assessment of both linear and quadratic trends over the three-day period.

#### 3.1.1 Condition

An overall main effect of Condition was found, F(3,153) = 3.24, *p* = 0.024. Specifically, regression analysis confirmed that participants in the Assortment (*β* = 1.07, *SE* = 0.45, *p* = 0.018) and Combined (*β* = 1.12, *SE* = 0.44, *p* = 0.011) conditions ordered significantly healthier meals compared to Control. The Feedback condition did not differ from Control (*β* = 0.29, *SE* = 0.43, *p* = 0.498).

#### 3.1.2 Day

There was a significant overall effect of Day, F(2,290) = 8.04, *p* < 0.001. While the linear trend was not significant (*β* = 0.59, *p* = 0.128), a significant quadratic effect of day, *β* = −1.18, *SE* = 0.37, *p* = 0.001, confirmed that healthy ordering increased from Day 1 to Day 2 but declined again on Day 3.

#### 3.1.3 Interaction

While the overall Condition × Day interaction was not significant (*p* = 0.162), the combined condition showed a significant interaction with the quadratic trend of Day, *β* = 1.09, *SE* = 0.52, *p* = 0.037, indicating an attenuation of the decline observed in the Control condition.

### 3.2 Meal component scores

**Figure 2:**
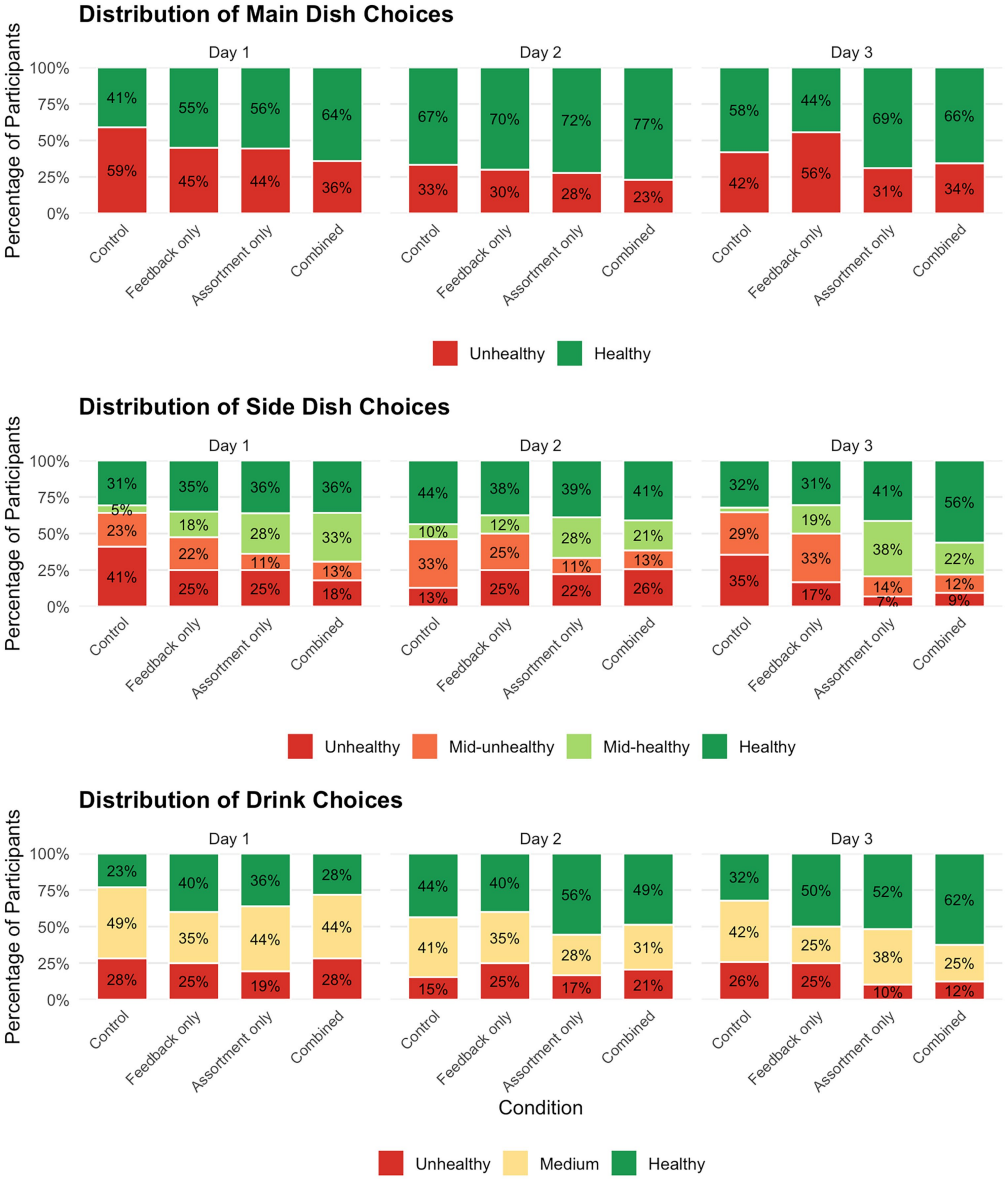
Distribution of dish choices by condition, day, and meal component. Stacked bars illustrate the percentage distribution of health scores for each meal component across conditions and study days. Health ratings were assigned as follows: main dishes (binary: 1 = unhealthy, 4 = healthy), side dishes (4 levels: 1 = unhealthy, 2 = mid-unhealthy, 3 = mid-healthy, 4 = healthy), and drinks (3 levels: 1 = unhealthy, 2.5 = medium, 4 = healthy).

Figure 2 displays the distribution of choices for main dishes, side dishes, and drinks across days and conditions, illustrating how the proportion of healthier versus less healthy choices shifted over time. The Combined and Assortment conditions showed the highest proportions of healthy choices across all components. The largest magnitude of sustained effect was observed in the Sides and Drinks for both the Assortment and Combined conditions. To analyze these trends, we utilized component-specific mixed-effects models: a Generalized Linear Mixed Model (GLMM) with a binomial distribution and logit link function was applied to the binary main dish choices (healthy vs. unhealthy). In contrast, Linear Mixed-Effects Models (LMM) were used for the side dishes and drinks, which were scored on four- and three-point scales, respectively.

#### 3.2.1 Main dishes

Only the Combined condition increased the likelihood of choosing a healthy main dish on Day 1 compared to the Control condition, *β* = 0.59, *SE* = 0.29, *p* = 0.040. This initial outperformance was subsequently attenuated as the proportion of healthy choices in the Control group increased between Day 1 and Day 2 to 66.7% and remained relatively high (58.1%) on Day 3. These healthy proportions approximated those of the Combined condition on Day 2 and Day 3 (76.9 and 65.6%, respectively). Day (*p* > 0.07) and Condition × Day effects were non-significant across conditions (*p* > 0.70).

#### 3.2.2 Side dishes

A significant main effect of Condition, F(3, 154) = 3.36, *p* = 0.020, confirmed that Assortment (*β* = 0.44, *SE* = 0.18, *p* = 0.018) and Combined (*β* = 0.49, *SE* = 0.18, *p* = 0.007) conditions’ participants selected healthier sides on average than the Control. As illustrated in Figure 2, these conditions showed a clear shift away from unhealthy side choices (scored 1) toward mid-healthy (scored 3) and healthy (scored 4) options, with the Combined condition reaching over 56% healthy side selections by Day 3. The significant quadratic component (*β* = −0.45, *SE* = 0.17, *p* = 0.010) confirmed an inverted U-shaped trend (decline after Day 2). Significant quadratic interactions for Assortment (*β* = 0.55, *SE* = 0.25, *p* = 0.028) and Combined (*β* = 0.70, *SE* = 0.24, *p* = 0.005) suggest that these nudges partially moderated the decline, leading to more stable healthy side choices relative to Control.

#### 3.2.3 Drinks

A significant overall quadratic effect of Day (*β* = −0.34, *SE* = 0.13, *p* = 0.009) indicated an inverted U-shaped trend (decline after Day 2). This decline was partially moderated in the Combined condition, as indicated by a significant positive linear interaction with Day, *β* = 0.42, *SE* = 0.19, *p* = 0.032. As shown in Figure 2, the proportion of healthy drink choices in the Combined condition increased steadily across all three days, reaching 62.5% on Day 3. In the Assortment condition, the proportion of healthy drink choices also increased slightly between Day 1 and Day 3 (36.1, 55.6, 51.7%, for Days 1–3, respectively), but the interactions were not significant (*ps*>0.20).

### 3.3 Additional variables

#### 3.3.1 Demographics

Mean daily health scores were similar for females (M = 8.52, SD = 1.96) and males (M = 8.32, SD = 2.03), with no consistent gender differences across conditions. Age was significantly associated with health scores (*β* = 0.05, *SE* = 0.02, *p* = 0.032), indicating that older participants generally made healthier choices. However, the interaction between age and condition was not significant, F(3, 150) = 0.60, *p* = 0.616.

#### 3.3.2 Internal states

To account for momentary physiological states, we re-ran the primary LMM including self-reported hunger as a covariate. Hunger was not a significant predictor of health scores (*β* = −0.15, *p* = 0.061), and its inclusion did not alter the main intervention effects. The Assortment (*β* = 1.03, *p* = 0.023) and Combined (*β* = 1.05, *p* = 0.018) conditions remained significant predictors of higher health scores compared to Control. Furthermore, the interaction between the Combined condition and the quadratic trend of Day remained significant (*β* = 1.12, *p* = 0.034), confirming that the nudge successfully sustained healthier choices over time even when adjusting for hunger levels. Similarly, self-reported mood was not a significant predictor of health scores (*β* = −0.02, *p* = 0.7) and did not interact significantly with experimental conditions, F(3, 376) = 1.44, *p* = 0.231.

## 4 Discussion

To our knowledge, this study is the first to examine the effects of digital nudges across repeated online ordering occasions. Our findings indicate that nudges combining just-in-time feedback with assortment design (combined condition) led to the most consistent improvements in meal healthiness over time. In contrast, the Assortment condition showed a strong trend albeit temporally less stable effects, and Feedback and Control participants maintained lower overall health scores. The repeated-measures design revealed that healthy ordering increased from Day 1 to Day 2 but declined by Day 3. This quadratic trend, observed even in the Control group, likely reflects novelty deterioration (23) and regression to the mean, where initial cognitive engagement with the ordering platform temporarily increases deliberate healthy choices before reverting to baseline dietary habits. From a behavioral economic perspective, this decline may also be driven by moral licensing (30), wherein healthy choices on Day 2 serve as a psychological justification for more indulgent ordering on Day 3. This decline was attenuated in the Combined condition, which by Day 3 was the only intervention to outperform the Control condition at the same time point, suggesting that the combination of structural (assortment) and moment-of-choice (feedback) nudges helps sustain healthier decisions over time. Our findings suggest that the Combined nudge is relatively robust across various participant states. However, the lack of significant moderation by factors such as hunger and mood may be specific to our experimental context and sample size. Future research using larger cohorts is needed to define the boundaries of these interventions’ external validity, as internal states often play a more volatile role in real-world, non-simulated food ordering.

Analysis by meal component showed that main dish choices, coded as healthy versus unhealthy, were more stable. While the Combined nudge established a significant immediate advantage on Day 1, this difference was attenuated over time as healthy proportions in the Control group increased to approximate the Assortment and Combined conditions by Day 3. In contrast, side dishes and drinks in the Assortment and Combined conditions exhibited continued growth or stability through Day 3, successfully counteracting the decline observed in the Control group. This stability could reflect participants’ stronger preferences or habits regarding main dishes, or the binary scoring structure that eliminated the possibility of incremental change. Side dishes, with four levels of healthiness, exhibited the most dynamic responses to nudges, while drinks, with three levels, showed intermediate responsiveness. These differences align with the idea that behavioral adjustments are easier when incremental improvements are possible (31). In addition, participants may have been more willing to shift side and drink choices gradually than to switch core meals entirely. In applied settings, this suggests that interventions targeting flexible, adjustable choices (like sides and beverages) may produce more rapid and visible effects on overall meal healthiness. Future studies should test whether the stability shown in main dish choices reflects strong pre-existing habitual preferences regarding main dishes, or the constraints of the available choice set, by offering a broader and more graded range of main dish options.

The practical contribution of this study lies in providing a specific strategy for digital food environment governance that balances public health goals with commercial feasibility. We propose that platforms can implement our findings through interface-level assortment standards rather than restrictive restaurant-level mandates. For instance, platforms could require a balanced ratio of healthy-to-unhealthy options in “Default Side” selections or “Recommended Add-ons” (Assortment), ensuring that healthier alternatives are as readily accessible as calorie-dense options. Regulators may require platforms to have a balanced ratio as parts of the terms of their license to operate as food vendors in the country. Furthermore, we suggest that platforms integrate real-time evaluative feedback at the basket level. By providing a dynamic healthiness score that updates as items are added (Feedback), platforms can leverage the temporal sustainability observed in our Combined condition. This dual-layered approach—optimizing the immediate choice set ratio while providing real-time feedback—allows platforms to support long-term healthy habituation without requiring a fundamental restructuring of restaurant menus or compromising algorithm-driven delivery logistics based on vicinity. Here too, regulators may state this as part of licensing requirements.

There were several limitations in this study. First, participants completed orders in a simulated platform, which may not fully capture real-world decision-making under time pressure or budget constraints. Second, the Control condition served as an active control, as it included labels marking healthy choices rather than being a full control group with no informational aids. Third, the binary distribution of main dishes may have limited participants’ ability to make gradual changes compared to sides or drinks, which had more variety of healthiness. Future studies should explore the development of more granular scoring systems for main dishes to allow for a more sensitive assessment of incremental behavioral shifts in primary meal choices. Finally, while the study followed participants across repeated decisions, three days is too brief to evaluate the longer-term sustainability of behavior change in everyday contexts such as food choices.

Future research should examine longer-term effects and evaluate real-world implementation to enhance effectiveness. Overall, this study provides practical, policy-relevant evidence for leveraging combined, complementary digital interventions to promote more effective public health within the digital food ecosystem.

## Data Availability

The raw data supporting the conclusions of this article will be made available by the authors, without undue reservation.
