# Supplementary material for: Beyond one-shot nudges: the effects of repeated interventions for healthier online meal ordering
Source: Front Nutr. 2026 May 4;13:1787724. doi: 10.3389/fnut.2026.1787724 (PMC13180568; doi:10.3389/fnut.2026.1787724)
Supplement: Supplementary file 1 [file Table_1.pdf]

Supplementary Material

1 Supplementary Table 1

Meal Component Health Scores

Condition	Control & Feedback		Assortment & Combined	
Type	Dish	Score	Dish	Score
Main	Chicken breast	4	Chicken breast	4
Main	Salmon fillet	4	Salmon fillet	4
Main	Sausages	1	Peppers stuffed with rice & legumes	4
Main	Fried fish	1	Roastbeef	4
Main	Schnitzel	1	Schnitzel	1
Main	Beef kebab	1	Beef kebab	1
Side	Green salad	4	Green salad	4
Side	Grilled vegetables	4	Grilled vegetables	4
Side	Mujaddara (rice & lentils)	3	Market salad	4
Side	Rice	2	Couscous with vegetables	3
Side	Grilled potatoes	2	Mujaddara (rice & lentils)	3
Side	Potato puree	2	Rice with beans	3
Side	French fries	1	Grilled potatoes	2
Side	Sweet potato fries	1	French fries	1
Side	Fried onion rings	1	Fried onion rings	1
Drink	Still water	4	Still water	4
Drink	Sparkling water	4	Sparkling water	4
Drink	Diet Coke	2.5	Diet Coke	2.5

Drink	Diet Sprite	2.5	Diet Sprite	2.5
Drink	Coke	1	Coke	1
Drink	Ice tea	1	Ice tea	1

Note. Health scoring was binary (healthy/unhealthy) for main dishes, while side dishes and drinks were scored on four- and three-point scales, respectively. In the Control and Feedback conditions, the ratio of healthy meal items relative to the total number of items presented was 1/3. In the Assortment and Combined conditions, the ratio of healthy items was 2/3.
